# Failure to achieve lupus low disease activity state (LLDAS) six months after diagnosis is associated with early damage accrual in Caucasian patients with systemic lupus erythematosus

**DOI:** 10.1186/s13075-017-1451-5

**Published:** 2017-11-10

**Authors:** Matteo Piga, Alberto Floris, Giulia Cappellazzo, Elisabetta Chessa, Mattia Congia, Alessandro Mathieu, Alberto Cauli

**Affiliations:** Chair of Rheumatology and Rheumatology Unit, University Clinic and AOU of Cagliari, SS 554, 09042 Monserrato, Cagliari Italy

**Keywords:** Systemic lupus erythematosus, Disease activity, Outcomes research

## Abstract

**Background:**

The aim was to assess the attainability and outcome of the lupus low disease activity state (LLDAS) in the early stages of systemic lupus erythematosus (SLE).

**Methods:**

LLDAS prevalence was evaluated at 6 (T1) and 18 (T2) months after diagnosis and treatment initiation (T0) in a monocentric cohort of 107 (median disease duration 9.7 months) prospectively followed Caucasian patients with SLE. Reasons for failure to achieve LLDAS were also investigated. Multivariate models were built to identify factors associated with lack of LLDAS achievement and to investigate the relationship between LLDAS and Systemic Lupus International Collaboration Clinics (SLICC)/Damage Index (SDI) accrual.

**Results:**

There were 47 (43.9%) patients in LLDAS at T1 and 48 (44.9%) at T2. The most frequent unmet LLDAS criterion was prednisolone dose >7.5 mg/day (83% of patients with no LLDAS at T1). Disease manifestations with the lowest remission rate during follow up were increased anti-double-stranded DNA (persistently present in 85.7% and 67.5% of cases at T1 and T2, respectively), low serum complement fractions (73.2% and 66.3%) and renal abnormalities (46.4% and 28.6%). Renal involvement at T0 was significantly associated with failure to achieve LLDAS both at T1 (OR 7.8, 95% CI 1.4–43.4; *p* = 0.019) and T2 (OR 3.9, 95% CI 1.4–10.6; *p* = 0.008). Presence of any organ damage (SDI ≥1) at T2 was significantly associated with lack of LLDAS at T1 (OR 5.0, 95% CI 1.5–16.6; *p* = 0.009) and older age at diagnosis (OR 1.05 per year, 95% CI 1.01–1.09; *p* = 0.020).

**Conclusion:**

LLDAS is a promising treatment target in the early stages of SLE, being attainable and negatively associated with damage accrual, but it fit poorly to patients with renal involvement.

## Background

Systemic lupus erythematosus (SLE) is a chronic multisystem disease, characterized by wide heterogeneity in clinical presentation, course and responsiveness to therapy [1, 2]. Despite significant advances in the understanding of the pathophysiology and optimization of medical care [3, 4], patients with SLE have an age-standardized mortality rate of 3.2 per 1 million people [5] and carry a risk of progressive organ damage accrual and reduced health-related quality of life [6, 7].

The treat-to-target (T2T) approach, successfully applied in rheumatoid arthritis and other non-rheumatic conditions, is a therapeutic strategy aiming to improve disease outcomes through the achievement of a pre-specified goal [8]. An international task force has recently suggested applying the T2T strategy in SLE, recommending that the treatment target should be remission or, where this cannot be reached, the lowest possible disease activity [9]. Many different definitions of remission, none of them generally accepted, are currently available in the literature and many controversial issues have to be clarified, such as its real attainability as a treatment target in clinical practice [10–12]. On the one hand, a framework for definition of remission in SLE (DORIS) has been recently set up by an international expert group [12]. On the other hand, a composite definition of minimal acceptable disease activity, the lupus low disease activity state (LLDAS), has been proposed by the Asia-Pacific Lupus Collaboration (APLC) [13]. LLDAS is based on the following criteria: (1) SLE Disease Activity Index 2000 (SLEDAI-2K) ≤4, with no activity in major organ systems (renal, central nervous system, cardiopulmonary, vasculitis, gastrointestinal, haemolytic anaemia fever); (2) no new lupus disease activity compared with the previous assessment; (3) Safety of Estrogen in Lupus Erythematosus National Assessment (SELENA)-SLEDAI physician global assessment (PGA) ≤1; (4) current prednisolone (or equivalent) dose ≤7.5 mg daily; and (5) well-tolerated standard maintenance doses of immunosuppressive drugs and approved biological agents [13].

Early damage was demonstrated to be associated with lower 10-year survival, such that patients with initial damage have a fourfold higher mortality rate compared to those with no early damage [14]. In order to be considered a valid treatment target, LLDAS should be proved as being protective against damage accrual in the early SLE stages. However, LLDAS prevalence and its association with damage have been investigated in a few longstanding SLE cohorts with no homogeneous disease duration [13, 15, 16] and therefore no data are currently available on its potential role as an early treatment target.

This study primarily aimed to assess the frequency of LLDAS achievement and its association with early damage accrual in a homogeneous cohort of Caucasian patients with SLE prospectively assessed during the first 18 months of treatment after diagnosis. The secondary aim was to identify the main reasons for failure to achieve LLDAS.

## Methods

### Patients

Data from patients included in the Cagliari (Italy) SLE cohort [17] between 1 January 2006 and 31 December 2016 were used for this study. Inclusion criteria were: (a) SLE diagnosed according to the revised 1997 American College of Rheumatology (ACR) criteria [18]; (b) starting the first treatment for SLE at enrolment in the Cagliari cohort; (c) moderate to severe disease activity (SLEDAI-2K ≥6) at baseline; (d) at least quarterly visits during the study interval; and (e) age ≥18 years.

Retrospective analysis of prospectively collected data was performed, reviewing medical records and clinical files. Baseline was fixed at the time of diagnosis, which corresponded to treatment initiation (T0). No specific recommendations are currently available on the appropriate time to assess the target achievement in a T2T strategy for SLE [9]. In order to assess LLDAS as a goal for initial treatment we set the primary study endpoint at 6 months (T1), according to the average induction therapy duration recommended for severe disease manifestations, such as glomerulonephritis [19]. Eighteen months (T2) was considered as an appropriate time to evaluate the effect of maintenance treatment and the early damage accrual [14, 20].

### Patient assessment

Demographic factors including gender, age at onset and diagnosis were collected. Disease onset was defined as the time of appearance of the first clinical classification criterion. Disease duration was the time period between disease onset and diagnosis. The number of 1997 ACR criteria presented from disease onset up to the baseline was recorded. At baseline, antinuclear antibodies (ANA) (tested by indirect immunofluorescence (IIF), using Hep2 cell substrate, positivity was defined as a titre >1:160), anti-Ro/SSA, anti-La/SSB, anti-Sm, anti-RNP, lupus anticoagulant, anticardiolipin (IgG nv <12 GPL; IgM nv <12 MPL) and anti-B2-glicoprotein1 (IgG nv <12 GPL; IgM nv <12 MPL) antibodies were assessed. Anti-dsDNA antibodies (by Farr assay; nv <10 IU/mL) and C3 (nv 90–180 mg/dL), C4 (nv 10–40 mg/dL) serum complement fractions were quarterly tested.

At each visit, disease activity was assessed using the SLEDAI-2K score and the PGA (0–3) [21]. Damage accrual was assessed at 18 months by the SDI [22] and the possible attribution to corticosteroids was done according to a previous definition [23]. Ongoing use and new prescription of corticosteroids, anti-malarial and immunosuppressant drugs (azathioprine, methotrexate, mycophenolate mofetil, cyclosporine A, cyclophosphamide or rituximab) were assessed at every visit. Average daily dose of prednisolone (or equivalent) was recorded for each patient at every study follow-up visit.

### Data analysis

Normally and non-normally distributed variables were summarized using mean ± standard deviation (SD) and median with interquartile range (IQR), respectively. Univariate and multivariate analysis were performed to evaluate potential factors associated with failure to achieve LLDAS. Univariate analysis was performed using the two-sample Student *t* test or Mann–Whitney test for quantitative variables, and the Chi-square test or Fisher's exact test for qualitative variables. Variables with a *p* value <0.1 on univariate analysis were included in a multivariate model for stepwise logistic regression. The odds ratio (OR) with 95% confidence interval (95% CI) was calculated.

To assess the association between lack of LLDAS achievement and damage development, a logistic regression model was created. Occurrence of any damage (SDI ≥1) at 18 months was included as the dependent variable, whereas age, disease duration, male gender, average daily steroids dosage, renal involvement, use of anti-malarial or immunosuppressant drugs [24] and failure to achieve LLDAS at T1 and at T2 comprised the independent variables. Statistical significance was set at a *p* value <0.05. MedCalc® statistical software (Mariakerke, Belgium) was used.

## Results

### Patients

Overall, 178 new patients joined the Cagliari (Italy) SLE cohort during the study interval. The study cohort consisted of 107 (60.1%). The relevant features of Caucasian patients with SLE who fulfilled the inclusion criteria for study enrolment are summarized in Table 1. Regarding excluded patients, 31 were diagnosed elsewhere and were already on treatment at the time of enrolment, 25 had no quarterly follow up, 7 were younger than 18 years and 6 had SLEDAI-2K <6 at baseline.

**Table 1:** Demographic, clinical and serological features at baseline

Demographic features	Value
Female gender, *n* (%)	96 (89.7%)
Caucasian, (%)	107 (100%)
Onset age, median (IQR) years	31.3 (25.0–42.6)
Age at diagnosis, median (IQR) years	34.3 (26.5–43.7)
Disease duration at diagnosis, median (IQR) months	9.7 (6.0–27.6)
ACR 1997 clinical criteria	
Malar rash, *n* (%)	29 (27.1%)
Discoid rash, *n* (%)	7 (6.5%)
Photosensitivity, *n* (%)	26 (24.3%)
Oral ulcers, *n* (%)	11 (10.3%)
Arthritis, *n* (%)	96 (89.7%)
Serositis, *n* (%)	32 (29.9%)
Renal disorders, *n* (%)	27 (25.2%)
Class V^a^	4 (3.7%)
Class IV^a^	8 (7.5%)
Class III^a^	6 (5.6%)
Class II^a^	3 (2.8%)
Not biopsy proven	6 (5.6%)
Neurologic disorders, *n* (%)	3 (2.8%)
Haematological disorders, *n* (%)	61 (57.0%)
Disease activity	
SLEDAI-2K score, median (IQR)	10.0 (8.0-15.8)
SLICC Damage Index, median IQR^b^	0 (0-0)
Serological features	
ANA, *n* (%)	106 (99.1%)
Anti-dsDNA, *n* (%)	78 (72.9%)
Anti-Sm, *n* (%)	19 (17.8%)
Anti-RNP, *n* (%)	27 (25.2%)
Anti-SSA, *n* (%)	46 (43.0%)
Anti-SSB, *n* (%)	13 (12.1%)
Any aPLs	26 (24.3%)
Treatment	
PDN dose at T0, median (IQR) mg/day	15.0 (6.5–26.9)
PDN dose T0–T2, median (IQR) mg/day	10.4 (5.7–18.2)
Anti-malarial drug, *n* (%)	67 (62.6%)
Immunosuppressant drug, *n* (%)	68 (63.5%)
Methotrexate, *n* (%)	24 (22.4%)
Cyclophosphamide, *n* (%)	21 (19.6%)
Azathioprine, *n* (%)	17 (15.9%)
Cyclosporine A, *n* (%)	5 (4.7%)
Mycophenolate mofetil, *n* (%)	2 (1.9%)
Rituximab, *n* (%)	1 (0.9%)

*ACR* American College of Rheumatology, *ANA* antinuclear antibodies, *aPLs* positivity for lupus anti-coagulant (LAC) and/or anticardiolipin and/or antiBeta2-GPI antibodies, *PDN* prednisolone (or equivalent), *SLEDAI-2K*, Systemic Lupus Erythematosus Disease Activity Index 2000, *SLICC* Systemic Lupus International Collaboration Clinics ()/SLICC

^a^According to the International Society of Nephrology/Renal Pathology Society (ISN/RPS) 2003 classification of lupus nephritis

^b^At diagnosis the SLICC Damage Index is equal to 0 by definition

### LLDAS achievement

**Fig. 1:**
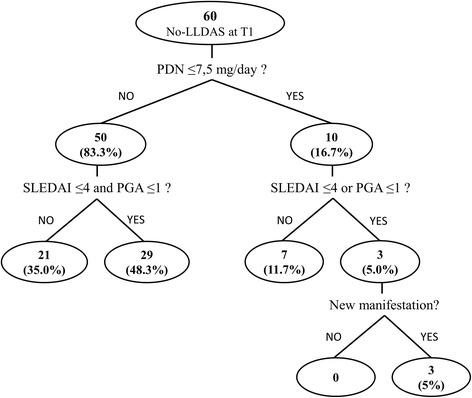
Analysis of the main causes of lack of lupus low disease activity state (LLDAS) achievement. SLEDAI-2K, Systemic Lupus Erythematosus Disease Activity 2000; PGA, physician global assessment; PDN, prednisolone (or equivalent)

At T1 (6 months), LLDAS was achieved by 47 (43.9%) patients. Focusing on unmet criteria for LLDAS in the remaining 60 (56.1%) patients: 50 (83.3%) were not on prednisolone ≤7.5 mg daily and 29 of them had SLEDAI-2K ≤4 and PGA ≤1; 7 (11.7%) did not have SLEDAI-2K ≤4 or PGA ≤1 with prednisolone dosage ≤7.5 mg/day, 3 (5.0%) experienced new manifestation (Fig. 1).

**Fig. 2:**
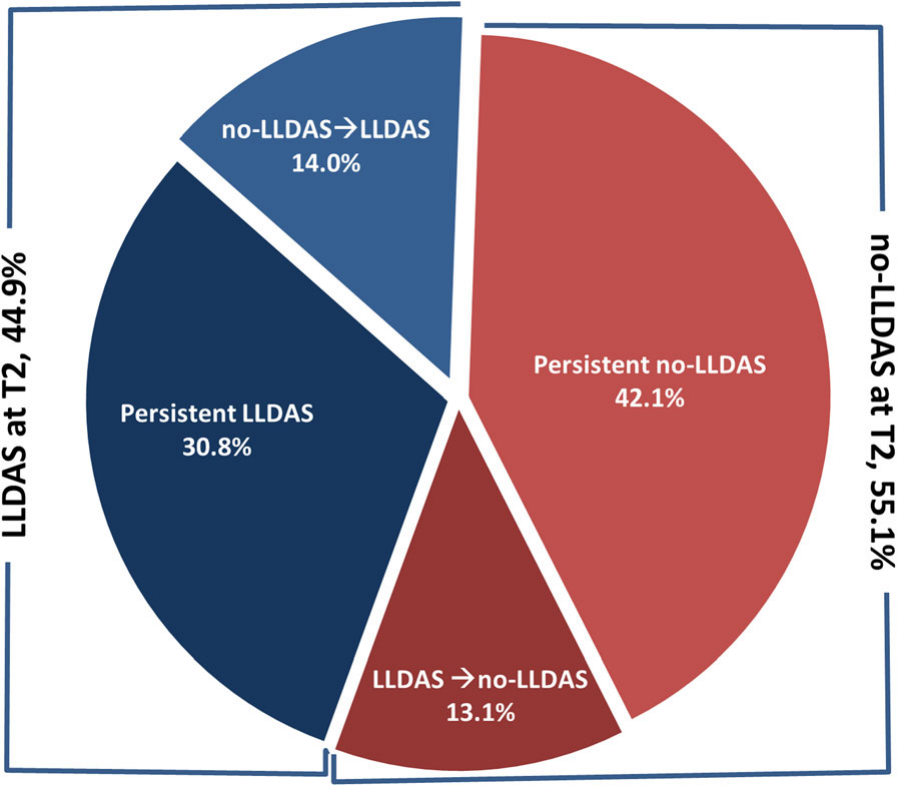
Patients who were in lupus low disease activity state (LLDAS) and patients who were not in LLDAS (no-LLDAS) at 18 months (T2). Persistent LLDAS, patients who were in LLDAS at 6 months (T1) and at T2; No-LLDAS → LLDAS, patients who achieved LLDAS within the T1–T2 interval; Persistent no-LLDAS, patients who never achieved LLDAS; No-LLDAS ← LLDAS, patients who were in LLDAS at T1 but not at T2

At T2 (18 months), 48 (44.9%) patients were in LLDAS; 33 of them achieved LLDAS at T1 and were still in this condition at T2, whereas 15 reached LLDAS within the interval between T1 and T2 for the first time. Out of 59 (55.1%) patients who were not in LLDAS at T2, 45 had never been in LLDAS and 14 were in LLDAS at T1 but no longer at T2 (Fig. 2). Major reasons for loss of LLDAS in these 14 patients were the onset of new disease activity manifestations in 7 (50.0%) patients (3 with articular, 3 with cutaneous and 1 with vasculitis manifestations), intolerance of drug treatment in 5 patients (35.7%) and a new finding of positivity for anti-dsDNA or low complement in 2 patients (14.3%).

### Persistently active SLEDAI items

**Fig. 3:**
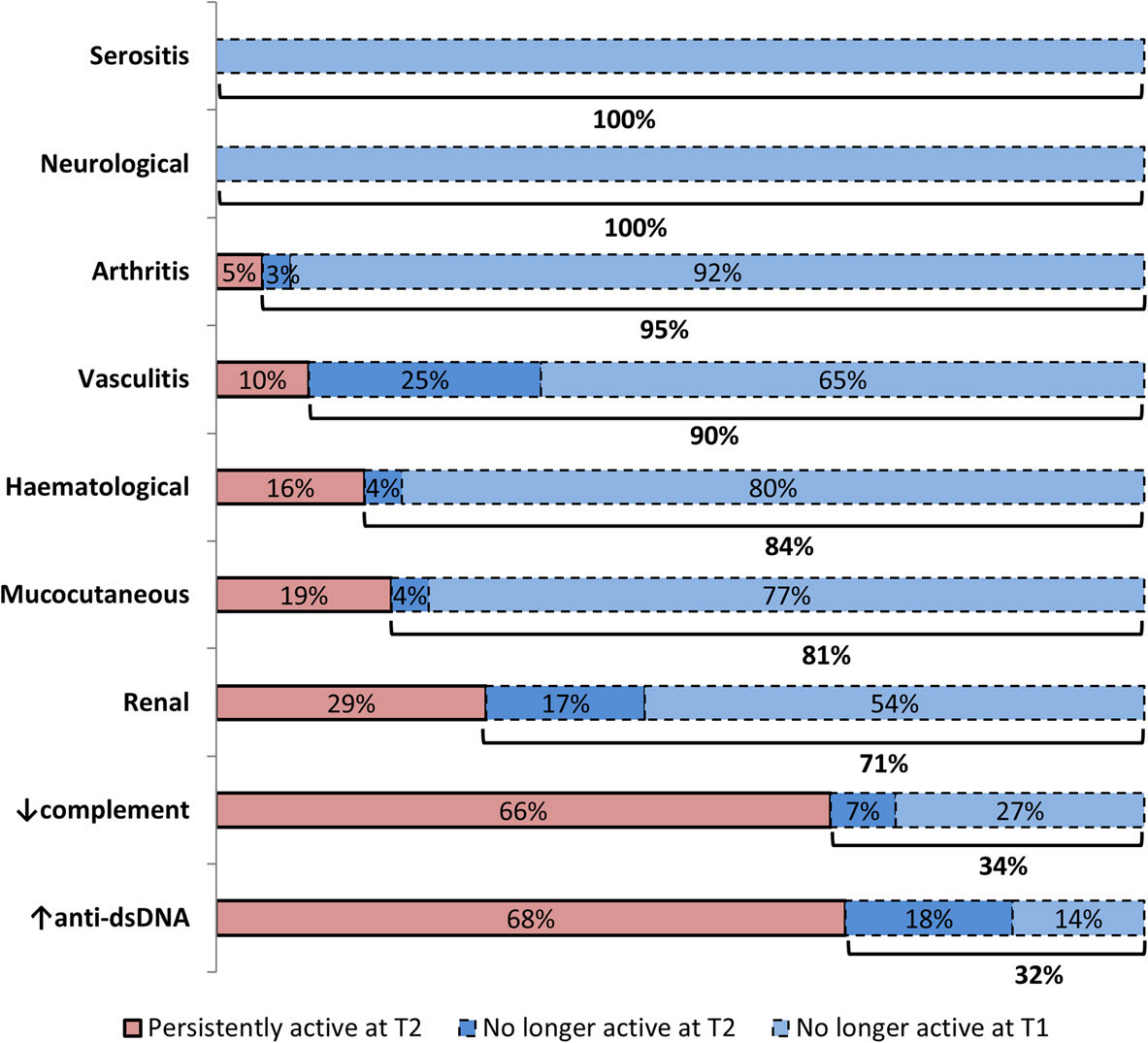
Frequency (percentage) of manifestations still identified at 18 months (T2) and respective remission rate. Purple, frequency (percentage) of manifestations recorded at T0 and still present at T2. Dark blue, manifestations that were in remission at 6 months (T1). Light blue, manifestations that were recorded as in remission for the first time at T2. Below the bars the global remission rate is reported

Individual SLEDAI-2K items were grouped into neurologic, vasculitis, arthritis, renal, myositis, mucocutaneous, serositis, low complement, increased anti-dsDNA and haematologic disorders. SLEDAI-2K items with worse remission rates were high anti-dsDNA concentration, complement consumption and renal abnormalities. Increased anti-dsDNA levels were still present in 85.7% and 67.5% of patients at T1 and T2 respectively, low complement in 73.2% and 66.3% of patients and renal disorders in 46.4% and 28.6% of patients (Fig. 3).

### Factors associated with failure to achieve LLDAS

On univariate analysis, the following factors recorded at baseline were significantly associated with failure to achieve LLDAS at T1: renal involvement (25 (41.7%) vs 2 (4.3%); *p* < 0.001)), higher SLEDAI-2K score (median 13.0 (9.0–18.0) vs 8 (7.2–10.0); *p* < 0.001)), positive (>10UI/mL) anti-dsDNA antibodies (49 (81.7%) vs 29 (61.7%); *p* = 0.013), lower serum C3 (median 65.5 (40.1–80.5) vs 82.0 (67.0–90.8) mg/dL; *p* = 0.002) and C4 values (10.0 (4.0–13.0) vs 13.0 (10.0–16.0) mg/dL; *p* = 0.013), higher prednisolone dose (median 25 (14–38) vs 8 (5–15) mg/day; *p* < 0.001) and immunosuppressant drug use (45 (75.0%) vs 23 (48.9%); *p* = 0.010). On multivariate analysis renal involvement (OR 7.8, 95% CI 1.4–43.4; *p* = 0.019) and C4 level (OR 0.91, 95% CI 0.83–0.99; *p* = 0.036) were confirmed to be associated with failure to achieve LLDAS (Table 2).

**Table 2:** Demographic, clinical and serological factors associated with failure to achieve LLDAS at T1

	Univariate analysis			Multivariate analysis	
	No LLDA at T1 (n = 60)	LLDAS at T1 (n = 47)	*p* value	Odds ratio	*p* value
Demographic features					
Male, *n* (%)	6 (10.0%)	5 (10.6%)	1.00		
Onset age, median (IQR) years	32.3 (25.7–43.0)	29.2 (24.4–39.8)	0.221		
Diagnosis age, median (IQR) years	36.9 (28.2–45.1)	31.2 (25.6–40.6)	0.152		
Disease duration, median (IQR) months	9.0 (5.0–12.3)	10.6 (6.1–30.6)	0.340		
Clinical features					
Malar rash, *n* (%)	15 (25.0%)	14 (29.8%)	0.580		
Discoid rash, *n* (%)	4 (6.7%)	3 (6.4%)	1,000		
Photosensitivity, *n* (%)	16 (26.7%)	10 (21.3%)	0.519		
Oral ulcers, *n* (%)	7 (11.7%)	4 (8.5%)	0.752		
Arthritis, *n* (%)	54 (90.0%)	42 (89.4%)	1.00		
Serositis, *n* (%)	19 (31.7%)	13 (27.7%)	0.653		
Renal disorder, *n* (%)	25 (41.7%)	2 (4.3%)	**<0.001**	7.8 (1.41–43.4)	**0.019**
Neurologic disorder, *n* (%)	3 (5.0%)	0	0.254		
Haematological disorders, *n* (%)	37 (61.7%)	24 (51.1%)	0.272		
SLEDAI-2K, median (IQR) score	13 (9.0-18.0)	8 (7.2–10.0)	**<0.001**	-	-
Serological features					
ANA, *n* (%)	59 (98.3%)	47 (100%)	1.00		
Anti-dsDNA, *n* (%)	49 (81.7%)	29 (61.7%)	**0.013**	-	-
Anti-dsDNA, median (IQR) UI/mL	114 (46–2069)	106 (31–191)	0.057		
Anti-Sm, *n* (%)	11 (18.3%)	8 (17.0%)	0.860		
Anti-RNP, *n* (%)	17 (28.3%)	10 (21.3%)	0.404		
Anti-SSA, *n* (%)	23 (38.3%)	23 (48.9%)	0.272		
Anti-SSB, *n* (%)	5 (8.3%)	8 (17.0%)	0.172		
Any aPLs, *n* (%)	15 (25.0%)	11 (23.4%)	0.971		
C3, median (IQR) mg/dL	65.5 (40.1–80.5)	82.0 (67.0–90.8)	**0.002**	-	-
C4, median (IQR) mg/dL	10.0 (4.0–13.0)	13.0 (10.0–16.0)	**0.013**	0.91 (0.83–0.99)	**0.036**
Therapy					
Anti-malarials drugs, *n* (%)	41 (68.3%)	26 (55.3%)	0.167		
Immunosuppressant drugs, *n* (%)	45 (75.0%)	23 (48.9%)	**0.010**	-	-
PDN dose, median (IQR) mg/day	25.0 (13.7–37.5)	8.0 (5.0–14.8)	**<0.001**	1.04 (0.99–1.1)	0.073

Boxes are empty where statistical analysis was not applied. In the present logistic regression model the Systemic Lupus Erythematosus Disease Activity Index 2000 (SLEDAI-2 k) score was removed

*LLDAS* lupus low disease activity state, *No LLDAS* failure to achieve LLDAS, *aPLs* positivity for lupus anticoagulant (LAC) e/o aCL e/o Beta2-GPI antibodies, *PDN* prednisolone (or equivalent)

Numbers in bold indicate statistical significance for *p* < 0.05

Demographic, clinical and serological variables at baseline were also analysed as potential predictors of lack of LLADS at T2. On univariate analysis renal involvement (21 (36%) vs 6 (13%); *p* = 0.016), discoid rash (7 (11.9%) vs 0; p = 0.007), any aPLs (10 (16.9%) vs 16 (33.3%); *p* = 0.016) and average prednisolone dosage (median 12.5 (6.7–21.6) vs 8.0 (5.0–14.4) mg/day; *p* = 0.0159) were associated with no LLDAS achievement at 18 months. On multivariate analysis, renal involvement was the only factor independently associated with failure to achieve LLDAS at T2 (OR 3.9, 95% CI 1.4–10.6; *p* = 0.008).

### LLADS and early damage

After 18 months since diagnosis (T2), 23 patients out of 107 (21.5%) had SDI≥1 (range 0-3). Overall, 27 SDI items of damage were recorded in 23 patients at T2: 9 were definitely steroid-related (7 with cataracts, 1 with osteoporosis with vertebral collapse or fractures, 1 with muscle atrophy), 8 possibly steroid-related (5 with cognitive impairment, 1 with angina or coronary disease) and 9 independent of corticosteroid use (2 with cerebrovascular accident, 2 with pleural fibrosis, 2 with pulmonary fibrosis, 1 with tissue loss, 1 with proteinuria >3.5 g/24 h, 1 with valvular disease, 1 with thrombosis, 1 with erosive arthritis and 1 with malignancy).

Out of 23 patients with SDI ≥1 at 18 months, 19 (82.6%) had not achieved LLDAS at T1 and 18 (78.3%) at T2. In the logistic regression model failure to achieve LLDAS at T1 (OR 5.0 95% CI 1.5–16.6; *p* = 0.009) and age at diagnosis (OR 1.05 per year; 95% CI 1.01–1.09; *p* = 0.020) were independently associated with the presence of any damage at T2 (Table 3).

**Table 3:** Analysis of factors associated with any damage (SDI ≥1) at 18 months

	Distribution of potential predictors of damage		Multivariate analysis	
	SDI ≥1 (n = 23)	SDI = 0 (n = 84)	Odds ratio	*p* value
No LLDAS at T1, *n* (%)	19 (82.6%)	41 (48.8%)	5.0 (1.5–16.6)	0.009
No LLDAS at T2, *n* (%)	18 (78.3%)	41 (48.8%)	-	-
Male gender, *n* (%)	3 (13.0%)	8 (9.5%)	-	-
Diagnosis age, median (IQR) years	39.4 (34.2–52.9)	31.9 (25.6–41.6)	1.05 (1.01–1.09)	0.020
Disease duration, median (IQR) months	9.9 (4.9–36.0)	9.6 (6.2–25.0)	-	-
Renal involvement, *n* (%)	9 (39.1%)	18 (21.4%)	-	-
PDN, mg/day median (IQR)	16.7 (11.0–23.2)	8.4 (5.0–16.7)	-	-
Anti-malarial drugs, *n* (%)	9 (39.1%)	57 (67.9%)	-	-
Immunosuppressant drugs, *n* (%)	17 (73.9%)	57 (67.9%)	-	-

*SDI* Systemic Lupus International Collaborating Clinic/Damage Index (SDI), *No LLDAS* failure to achieve the lupus low disease activity state, *PDN* prednisolone (or equivalent)

## Discussion

In our study cohort 44% and 45% of patients were in LLDAS 6 and 18 months after treatment initiation, respectively. Despite a seemingly overall stable LLDAS rate, the dynamic nature of this condition was demonstrated. In fact, 14% of subjects first achieved LLDAS between the 6^th^ and 18^th^ month, whereas 13% of patients who were in LLDAS after 6 months of treatment lost LLDAS within the following 12 months. Golder et al. found that in a in a cross-sectional assessment, 44% of 1846 patients with SLE and a mean disease duration of 8.6 years did not achieve LLDAS [15]. Franklyn et al., in an SLE cohort with mean disease duration of 7.1 years, observed that 88% of patients achieved LLDAS at least once and that 38% of patients had this status for more than half of the follow-up duration [13]. These data suggest that the minimal disease activity, according to the LLDAS criteria, would be a more applicable goal in a T2T strategy than remission, according to the current definition [12, 25], especially in early disease stages [10]. Actually, Wilhem et al. observed that the median time to achieve durable remission, classified in four subtypes according to the principle proposed by the DORIS group, ranged between 1.8 and 11.0 years [10].

In this study, the most frequent reason for failure to achieve LLDAS 6 months after therapy initiation was daily prednisolone dosage >7.5 mg (83% of no LLDAS). In the study by Golder et al., prednisolone dosage was an unmet criterion in 57% of patients who did not achieve LLDAS [15]. In a study aiming to test the construct validity of LLDAS compared to expert opinion, disagreement was mainly due to low disease activity classification of patients with prednisolone daily dosage ≥7.5 mg (up to 10 mg/day), suggesting that such a higher dose was deemed acceptable by several experts who were interviewed [26]. Nevertheless, the lowest corticosteroid dose to be considered safe has not been definitely identified. Prednisolone >5 mg/day was reported to be associated with greater risk of osteoporosis, infections, cataracts and metabolic and cardiovascular disorders [27–29]. Tharmer et al. observed that the risk of damage accrual in patients with SLE did not substantially increase with prednisolone cumulative doses <180 mg/month (equivalent to 6 mg/day) [30]. In our cohort, damage was definitely attributable to steroid use in 40% of cases. Gladman et al. reported that 58% and 80% of the damage accrued in the first year after diagnosis and in later disease, respectively, could be described as “possibly or definitely” steroid-related [23]. In contrast, a larger proportion of early damage was related to early inflammatory organ damage [24]. In our study we found that failure to achieve early LLDAS (at 6 months) and older age at diagnosis, but not corticosteroid daily dose, were independently associated with presence of damage after 18 months. Two further retrospective studies reported that patients with LLADS had a reduced risk of damage accrual in at least half of observations [13, 16]. Supported by our data and literature evidence on damage development, we consider 7.5 mg/day an acceptable cutoff to define low disease activity during initial treatment, but A lower cutoff should be targeted to minimize risk of steroid-related damage during maintenance therapy in patients with SLE.

Renal involvement and serological disorders, consisting of low complement and increased anti-dsDNA, had the lowest remission rate in our cohort. However, renal involvement at baseline was the most important factor associated with failure to achieve LLDAS at 6 months (OR 7.8, 95% CI 1.4–43.4; *p* = 0.019) and 18 months (OR 3.9, 95% CI 1.4–10.6; *p* = 0.008) from treatment initiation. In a cross-sectional study, increased anti-dsDNA (OR 0.65, 95% CI 0.53–0.81; *p* < 0.001), renal involvement (OR 0.60, 95% CI 0.48–0.75; p < 0.001), low complement (OR 0.52, 95% CI 0.40–0.67; *p* < 0.001) shorter disease duration (OR 0.31, 95% CI 0.19–0.49; *p* < 0.001) and history of discoid rash (OR 0.66, 95% CI 0.49–0.89; *p* = 0.006) were identified as negatively associated with LLDAS [15]. These findings suggest that renal involvement and serological abnormalities are associated with a lower likelihood of LLDAS achievement.

Whether or not serological disorders should be considered in the definition of minimal disease activity it is a matter of debate [26]. Persistently increased anti-dsDNA and low complement may be part of the serological active clinically quiescent (SACQ) disease pattern [31], with some evidence suggesting a proportion of patients with SACQ disease can spend years without flares [32] whilst other may relapse [33]. However, increasing-fluctuating anti-dsDNA level rather than absolute values can predict disease flares [34]. Thus, in order to enhance the association between minimal acceptable disease activity and long-term outcomes, it would be conceivable to consider rising levels of anti-dsDNA antibodies rather than their steady positivity in the LLDAS definition.

This study has some limitations. First, the relatively small sample size may have hampered the study results. Nevertheless, by enrolling consecutively diagnosed patients at the time of treatment initiation and following them up prospectively, we added some novel information on LLDAS as a potential treatment target. Second, the retrospective design of the study prevented us from testing LLDAS criterion validity by comparing it with other treatment targets such as the SLE Responder Index [35].

## Conclusion

In conclusion, LLDAS is a promising treatment target in SLE, being attainable and negatively associated with damage accrual in the early stages of disease. However, it seems to poorly fit with the heterogeneity of clinical presentation in patients with SLE, mostly in those with renal involvement. On the one hand, the poor LLDAS attainability in patients with renal involvement could be due to greater severity and lasting disease activity in the renal domain. On the other hand, it could be due to the unsuitability of LLDAS in capturing the minimal disease activity in patients with renal involvement. Finally, greater consensus should be reached on the definition of serological disorders and prednisolone dose to be included in a unique definition of minimally acceptable disease activity.

## Abbreviations

ACR: American College of Rheumatology
ANA: Antinuclear antibodies
aPLs: Antiphospholipid antibodies
CI: Confidence interval
DORIS: Definition of remission in SLE
IIF: Indirect immunofluorescence
IQR: Interquartile range
LLDAS: Lupus low disease activity state
nv: Normal value
OR: Odds ratio
PGA: Physician global assessment
SACQ: Serological active clinically quiescent
SD: Standard deviation
SDI: Systemic Lupus International Collaboration Clinics/Damage Index
SELENA: Safety of Estrogen in Lupus Erythematosus National Assessment
SLE: Systemic lupus erythematosus
SLEDAI-2 K: Systemic Lupus Erythematosus Disease Activity Index 2000
SLICC: Systemic Lupus International Collaboration Clinics
T2T: Treat to target
